# Differential Equation-Based Prediction Model for Early Change Detection in Transient Running Status †

**DOI:** 10.3390/s19020412

**Published:** 2019-01-20

**Authors:** Xin Wen, Guangyuan Chen, Guoliang Lu, Zhiliang Liu, Peng Yan

**Affiliations:** 1 Key Laboratory of High-efficiency and Clean Mechanical Manufacture of MOE, National Demonstration Center for Experimental Mechanical Engineering Education, School of Mechanical Engineering, Shandong University, Jinan 250061, China; sduwenxin@mail.sdu.edu.cn (X.W.); chenguangyuan@mail.sdu.edu.cn (G.C.); yanpeng@sdu.edu.cn (P.Y.); 2 School of Mechatronics Engineering, University of Electronic Science and Technology of China, Chengdu 611731, China; zhiliang_liu@uestc.edu.cn

**Keywords:** prediction model, early change detection, differential equation, machine running status

## Abstract

Early detection of changes in transient running status from sensor signals attracts increasing attention in modern industries. To achieve this end, this paper presents a new differential equation-based prediction model that can realize *one-step-ahead* prediction of machine status. Together with this model, an analysis of continuous monitoring of condition signal by means of a null hypothesis testing is presented to inspect/diagnose whether an abnormal status change occurs or not during successive machine operations. The detection operation is executed periodically and continuously, such that the machine running status can be monitored with an online and real-time manner. The effectiveness of the proposed method is demonstrated using three representative real-engineering applications: external loading status monitoring, bearing health status monitoring and speed condition monitoring. The method is also compared with those benchmark methods reported in the literature. From the results, the proposed method demonstrates significant improvements over others, which suggests its superiority and great potentials in real applications.

## 1. Introduction

Real-world systems are seldom in steady status, and they almost always operate in transient conditions that are varying over time. Detection of change(s) in running status at an early stage enables to find and locate abnormal and unexpected/undesired system behavior(s) in its successive operations. With this, corrective schedule could be made to prevent potential operation failures and ensure equipment and product reliability, safety, quality, productivity, etc. [1,2,3,4]. The problem of running status monitoring may arise in process monitoring and control applications where the system needs to make a response and/or take appropriate actions as soon as possible after a change of machine status occurs [5,6,7,8].

In industrial and manufacturing areas existing approaches of change detection are mainly data-driven methods [7,9,10,11,12,13,14,15,16,17,18,19,20,21,22,23,24,25]. They normally use a detailed mathematical model evaluating dynamic running status, parameters of which are estimated and updated with relevant condition monitoring (CM) signals that are collected form considered machines. Once the dynamic machine behaviors are modeled, it is possible to detect potential status change(s) via analysis of changes of input parameters to the model.

Depending on the adopted models, these data-driven methods can be further classified into three categories: (1) density estimation methods [9,10,11,12], (2) rule-based or case-based methods [13,14,15,16] and (3) time-series analysis based methods [7,17,18,19,20]. The density estimation methods are developed from the availability of input/output histories of machine conditions that were collected from the field or laboratory. They are based on density estimation techniques modeling the past normal machine status, and produce change decision output based on the Bayesian probability theory for newly observed data. Typical Gaussian kernels [9] as well as relatives such as non-Gaussian process [11] and Gaussian mixture model (GMM) [12] can be employed to accomplish the density estimation. In spite of their success and popularity in simulation scenarios, they are, however, not effective when the available condition data is insufficient. The rule-based or case-based methods are expert systems that are driven by data mining, including the Dempster-Shafer (DS) theory [15], fuzzy set theory [16], etc. In these methods, there exist lots of parameter settings that need to be confirmed under human-made supervision, which would limit their practical value in real applications. In comparison, the time-series analysis methods use previous outputs regressed on to itself to provide an estimate of the current output, such that one-step-ahead prediction is allowed for residual error analysis in order to generate final detection result [19,20]. In the context of time-series analysis methods, various auto-regressive (AR) models have been proposed, going from simple linear time-series model to advanced nonlinear regression methods. For example, in [7], a linear AR model was assumed for analytical expression of those linear distributed condition signals, e.g., power consumption signal. Meanwhile, it has been widely recognized and accepted those periodic condition signals, such as vibration and sound signals, are more powerful when describing the machine system [21,22,23,24,25]. It is concluded that the statistical modelling by the way of AR models is relatively simplified in implementation, such that they can be applied to the monitoring and prognosis of industrial systems, especially when real-time and online processing is required.

In this paper, we extend the work of [26] and present a new prediction model with the purpose of monitoring and diagnosing the running status for a considered machine. The baseline of the presented method is an early change detection using a differential equation (DE) based prediction model. This model can describe dynamic properties of a system which change with time quite well and can predict the future status of the system conveniently [27,28]. Moreover, it has been also proven more powerful when discovering of sciential laws for dynamic systems [27]. The DE model has been successfully used in many fields [29,30,31,32]; however, to the best of our knowledge, it is the first time to apply it to machine running status monitoring. In this paper, together with the DE model, an analysis of continuous monitoring of CM signals by means of a null hypothesis testing is proposed to inspect/diagnose whether an abnormal running status change occurs or not in successive machine operations.

Besides of the methodological contributions put forth in the proposed method, we apply the method to three representative applications: external loading status monitoring, bearing health status monitoring, and speed condition monitoring. Three testing data sets are considered in the experiment. The first one is taken from the publicly available data set provided by the Case Western Reserve University (CWRU) [33], the second one is from the publicly available PRONOSTIA dataset provided by FEMTO-ST in France [34,35] and the last one is established based on our experimental setup. The proposed method is also compared with those reported in the literature, including autoregressive-integrated-movingaverage (ARIMA) model [18], root mean square (RMS) and kurtosis [36,37,38]. Experimental results demonstrate its significant improvements over others.

The rest of this paper are organized as follows. Section 2 describes the proposed prediction model. Section 3 gives the hypothesis testing used for decision-making. Section 4 gives the proposed framework for machine running status monitoring, followed by experiments in Section 5. Section 6 finally summarizes this paper with some concluding remarks.

## 2. Differential Equation-Based Prediction Model

This section first provides an overview of the proposed prediction model, and then discusses techniques carried out in the main steps.

### 2.1. Overview of the Proposed Model

Given a data stream composing of continuously monitoring condition signal $x_{t}$, let us assume that it has been denoted by a periodic form $x_{t}\to X_{nT+v}$, where *T* is the period, v∈[1,T] is the phase or timing, and *n* is the cycle index. In this paper, we assume the condition signal is periodically stationary as made in some previous works, e.g., [25,39], thus we can employ a prior periodicity estimation proposed in [18] to confirm the value of *T*. Then, main steps of the proposed prediction model includes the following:

- *Model formulation*, i.e., the proposed model is formulated with the considered CM signals. In this paper, a family of new time series are formed by arranging the original data at the same phase. As such, the model is formulated so as to predict next value of each phase.
- *Parameters estimation*, which estimates the parameters of the model. The numerical solution method of differential equations is used to estimate the model’s parameters. The parameters of the model are constantly updated during each data prediction process.
- *Data prediction*, i.e., the prediction of next data with the estimated model. The prediction value at each phase can be obtained with the model whose parameters have been estimated successfully.

The above steps are executed continuously for each observed cycles of CM data, allowing for residual error analysis that can quantify the error between the predicted values and the observed ones. The resulting error reflects the amount of temporal fluctuations in the CM data; more specifically, the higher residual error implies a higher probability that a change occurs and vice versa.

In the following, techniques used in the main steps of the model are discussed.

### 2.2. Proposed Model Description

#### 2.2.1. Model Formulation

For an arrived CM data stream up to the *k*th cycle, for each phase *v*, we take *i* continuous cycles before *k* to form a new series, i.e., $P_{v}=\left\{x_{(k-i)T+v},\ldots ,x_{(k-1)T+v}\right\}$, such that the DE model can be formulated for all phases.

Conventionally, the DE model relies on the following system state function f(·) to establish the prediction,

(1) $$\frac{dx_{t}}{dt}=f\left(x_{t},\beta ,t\right),$$

where $x_{t}$ is the value of differential equation in a series of time stamp *t*, β is parameter of the differential equation.

In this paper, since we use the past observed data at the same phase for prediction, the above-given new time series $P_{v}$ allows linear characteristics to emerge, based on which, we can obtain the prediction value, i.e., ${\hat{x}}_{(k)T+v}$ with the $P_{v}$ correspondingly. In the following, for each considered phase *v*, we re-denote the $P_{v}$ as the series $\left\{x_{t_{j}}\right\}$, j=1,…,i for simplicity.

According to the linear assumption made already, the time-series $\left\{x_{t_{j}}\right\}$ will satisfy,

(2) $$f(x_{t},\beta ,t)=a+b\times t,$$

where a(≠0) and b(≠0) are the parameters of differential equations that need to be estimated before prediction.

#### 2.2.2. Data Prediction

Assuming that we have estimated the values of *a* and *b*, according to [40], the corresponding time response function of Equation (1) can be written as

(3) $${\hat{x}}_{(k)T+v}=x_{t_{i+1}}=\frac{1}{2}b\times \left(t_{i+1}^{2}-t_{i}^{2}\right)+a\times \left(t_{i+1}-t_{i}\right)+{\bar{x}}_{t_{i}},$$

where $t_{i}=t_{0}+i\times h$, $t_{0}=0$, $t_{i+1}$ is the time stamp of the value $x_{t_{i+1}}$, which can also be regarded as the prediction value ${\hat{x}}_{(k)T+v}$, and *h* is the distance between the two time stamps. We set *h* as 1 in this article. The ${\bar{x}}_{t_{i}}$ is the initial value of the response function, represented by the average value of the sequence value in order to decrease effect of errors in data collection.

#### 2.2.3. Parameters Estimation

For the purpose of prediction of ${\hat{x}}_{(k)T+v}$, the first step is to estimate the optimal parameters of *a* and *b* in the DE model given in Equation (2). Because the signal is usually sampled through observation, we re-define the above variables at sampling time $t_{j}$ (j=1,…,i) with subscription *j* (e.g., $x_{t_{j}}=x_{j}$) for convenience in the following of this paper. The differential Equation (1) is calculated by simpsons method [40] by

(4) $$x_{j+2}-x_{j}=\frac{1}{3}h\left(f_{j+2}+4\times f_{j+1}+f_{j}\right)+R_{j+2},$$

in which $f_{j}=f\left(x_{j},u,t_{j}\right)$, j=1,…,i−2 and $R_{j+2}$ is local truncation error [40]. According to Equation (2), Equation (4) can be transformed into

(5) $$x_{j+2}-x_{j}=2\times a+\frac{h}{3}\left(t_{j+2}+4\times t_{j+1}+t_{j}\right)+R_{j+2},$$

which is thus re-written as

(6) $$\frac{x_{j+2}-x_{j}}{2}=a+\frac{h}{6}\left(t_{j+2}+4\times t_{j+1}+t_{j}\right)+\frac{R_{j+2}}{2}.$$

Then, Equation (6) can be re-expressed as

(7) $$x_{j+2}^{\left(0\right)}=a+x_{j+2}^{\left(1\right)}\times b+E_{j+2},$$

where $x_{j+2}^{\left(0\right)}=\frac{x_{j+2}-x_{j}}{2}$, $x_{j+2}^{\left(1\right)}=\frac{(t_{j+2}+4\times t_{j+1}+t_{j})h}{6}$, $E_{j+2}=\frac{R_{j+2}}{2}$. When $\sum_{j=1}^{i-2}E_{j+2}^{2}$ has a minimum value for the observed value $x_{j}$, the estimated parameters *a* and *b* can be finally obtained by

(8) $${\left(a,b\right)}^{T}={\left(B^{T}B\right)}^{-1}B^{T}Y,$$

in which

$$B={\left[\begin{array}{ccc} 1 & \cdots & 1\\ x_{3}^{\left(1\right)} & \cdots & x_{i}^{\left(1\right)} \end{array}\right]}^{T},Y={\left[\begin{array}{ccc} x_{3}^{\left(0\right)} & \cdots & x_{i}^{\left(0\right)} \end{array}\right]}^{T}.$$

The predicted value ${\hat{x}}_{(k)T+v}$ can be obtained by substituting the estimated parameters *a* and *b* into the time response Equation (3), where i=4 is used in the following experiment.

### 2.3. Residual Error Analysis

The residual error $q_{(k)T+v}$ can be defined by the absolute value of the difference between prediction data ${\hat{x}}_{(k)T+v}$ and the actual monitoring data $x_{(k)T+v}$ as

(9) $$q_{(k)T+v}=\left|{\hat{x}}_{(k)T+v}-x_{(k)T+v}\right|.$$

The cumulative value of $\left\{q_{(k)T+v}\right\}$ for a whole cycle is gathered, allowing for analyzing the machine condition periodically, which is calculated by

(10) $$Q_{k}=\sum_{v=1}^{T}q_{(k)T+v},$$

where $Q_{k}$ is the resulting value for the *k*th cycle.

Subsequently, we further standardize the obtained sequence $\left\{Q_{k}\right\}$ by $s_{k}=\left(Q_{k}-\bar{Q}\right)/\sigma$, where $\bar{Q}$ and σ are the sample mean and standard deviation of $\left\{Q_{k}\right\}$.

The anomaly score $\left\{s_{k}\right\}$ describes the dynamic characteristics of machine condition monitoring over time. That is, when the condition of machine is stable, this implies that the condition change does not occur, and the values of $\left\{s_{k}\right\}$ will be relatively small; otherwise, the values will be large.

### 2.4. Simulation Validation

**Figure 1:**
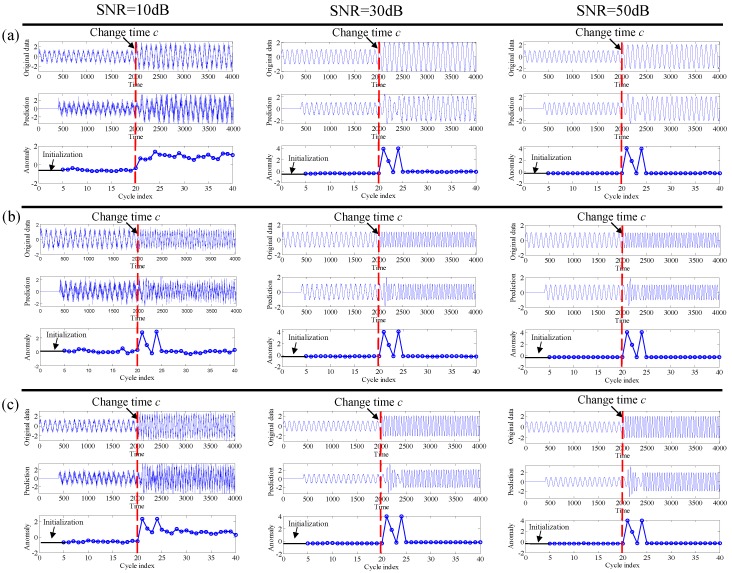
Results on testing data containing different changes: (**a**) amplitude change; (**b**) frequency change; (**c**) amplitude and frequency change.

For the purpose of evaluation of the effectiveness of the proposed model, we applied it on synthetically generated testing data, where different levels of Gaussian White Noise are added for simulating the real engineering scenarios. Considering that the status change(s) in real machine operations can result in the change(s) of relevant CM variables in terms of amplitude, frequency, or both of them [7,18,41,42,43], the testing signals are formulated, respectively, as

(11) $$x_{t}=\left\{\begin{array}{cc} sin(\omega \times t)+noise, & 1\leq t\leq c-1,\\ 2\times sin(\omega \times t)+noise, & c\leq t\leq m, \end{array}\right.$$

(12) $$x_{t}=\left\{\begin{array}{cc} sin(\omega \times t)+noise, & 1\leq t\leq c-1,\\ sin(2\omega \times t)+noise, & c\leq t\leq m, \end{array}\right.$$

(13) $$x_{t}=\left\{\begin{array}{cc} sin(\omega \times t)+noise, & 1\leq t\leq c-1,\\ 2\times sin(2\omega \times t)+noise, & c\leq t\leq m, \end{array}\right.$$

where *c* is the change time, *m* is the length of data, and ω is set as π. The results are given in Figure 1, where the Figure 1a–c shows the simulated signals generated by Equations (11)–(13), respectively. For each subfigure, the SNR are set as 10, 30 and 50 dB, respectively. It can be clearly seen that the anomaly scores are stable before the status change, and then the occurrence of status change causes an abrupt increase of the anomaly score, which implies that it can be detected successfully. Here, we also found that, for amplitude change and amplitude and frequency change cases, the residuals of 30 dB and 50 dB are different from the one of the case 10 dB. The main possible reason is that, with a lower SNR, i.e., 10 dB, the noise will be more taken into account for prediction of the future value with an amplitude change; however, with a higher SNR, the noise will have less influence on the perdition process.

## 3. Hypothesis Testing for Decision-Making

On the basis of the resulting anomaly score $s_{k}$, we test a null hypothesis using the 3σ criterion in order to detect whether a change occurs at the current *k*th cycle by

(14) $$\begin{array}{c} H_{0}:|s_{k}-{\bar{s}}_{k-1}|<3\sigma^{\prime },\\ H_{1}:|s_{k}-{\bar{s}}_{k-1}|\geq 3\sigma^{\prime }, \end{array}$$

where $H_{0}$ means that no change occurs on the *k*th cycle as long as $|s_{k}-{\bar{s}}_{k-1}|<3\sigma^{\prime }$, and $H_{1}$ indicates that a change occurs when $|s_{k}-{\bar{s}}_{k-1}|\geq 3\sigma^{\prime }$ (Note that the beginning nine cycles of data are used for initialization and the change detection begins with the 6th cycle after initialization). Here, ${\bar{s}}_{k-1}$ and $\sigma^{\prime }$ are the mean and standard deviation of an assumed Gaussian distribution, respectively, and they are calculated by

(15) $${\bar{s}}_{k-1}=\frac{1}{k-1}\sum_{j=1}^{k-1}s_{j},$$

(16) $$\sigma^{\prime }=\sqrt{\frac{1}{k-1}\sum_{j=1}^{k-1}{\left(s_{j}-{\bar{s}}_{k-1}\right)}^{2}}.$$

Here, it is worth mentioning that there exist other alternatives such as Gaussian Mixed Model (GMM) [44] and other non-Gaussian assumptions [11,45] for change detection. However, since the focus of this paper is on the prediction model, we do not investigate these alternatives in this study.

## 4. Proposed Machine Running Status Monitoring Framework

Together with the DE model described in Section 2, an analysis of continuous monitoring of CM signals by means of a null hypothesis testing (Section 3) is proposed to inspect/diagnose whether an abnormal running status change occurs or not in successive machine operations. Total four steps are included in the framework as given below:

(1) Collect CM data from the considered machine in a continuous manner;
(2) Compute the prediction value using the proposed DE model;
(3) Calculate anomaly scores based on residual error analysis at the current inspection time;
(4) Make the change decision by testing a null hypothesis. Report an alarm to the user; otherwise, go to Step 2 to continue.

**Figure 2:**
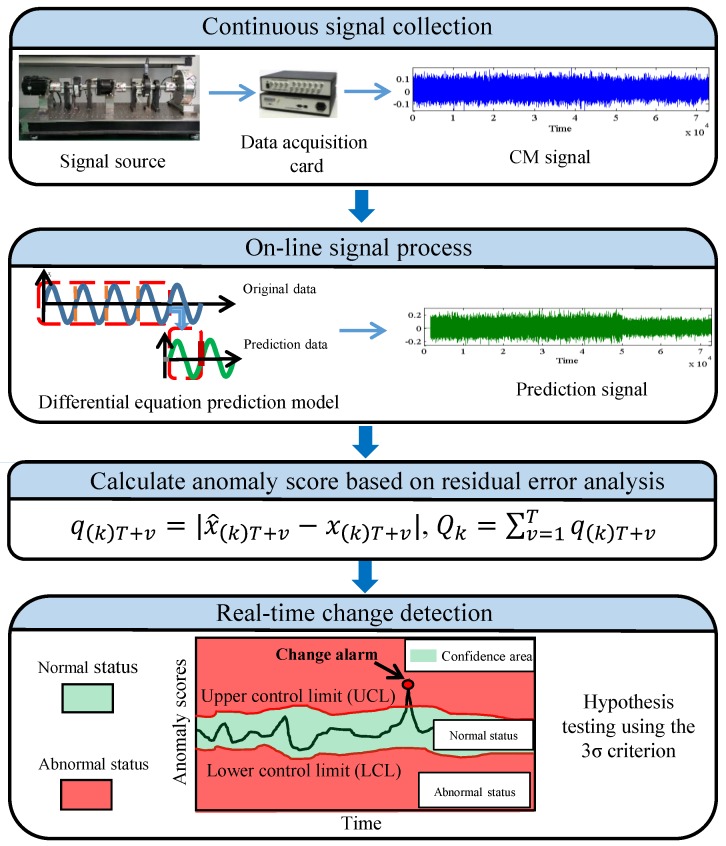
Flowchart of the proposed framework.

The flowchart of the proposed framework is provided in Figure 2.

## 5. Experimental Validation

To evaluate the effectiveness of the proposed method, we applied it to three representative industrial applications/tasks which are listed as below:

- *External loading status monitoring*: External loading status is essential for condition monitoring during unsteady machine operations because a piece of equipment under operation may be exposed to a series of varying loads according to the user’s needs [46]. Moreover, the load is a critical operating condition factor which has significant impact on machine health [47]. Detection of changes in load condition makes it possible for the machine to adjust itself once a load change occurs for safety protection [48].
- *Bearing health status monitoring*: As we all know, functional degeneration of machine components during the lifetime is common and unavoidable. The component degeneration will cause some undesired/unexpected consequences [48,49,50]. Based on CBM, maintenance can be scheduled in an optimal way with respect to cost, reliability, availability, or other logistic metrics of interest. Thus, automatic detection of changes in bearing health status can serve as a starting point for fault diagnosis or prediction of functional failure at an early stage.
- *Rotational speed monitoring*: The rotational speed in machine operations may fluctuate due to condition variations or unsteady environments [51]. Speed condition monitoring helps to find the unexpected running behaviors for operation maintenance [52,53], thus highly desired in online process monitoring of industrial manufacturing, numerical controlled machining, ect.

The proposed method is compared with the ARIMA model proposed in [18]. In addition, we also compare it with two methods: RMS and kurtosis, which are widely used in the literature [36,37,38]. In the following experiments, we utilize the first detected alarm to determine whether the detection is successful or false. We then use the precision indicator which is defined as ratio of the number of correctly detected changes over the total number of changes to quantify the detection performance. However, for the case only including several testing data, we directly provide the detection result.

### 5.1. Case Study I: External Loading Status Monitoring

The testing data used in this section are the motor bearing data provided by CWRU [33]. In the experiment, the vibration data were collected from the drive end and the fan end of the motor driving mechanical system with the sampling frequency of 12 kHz [54].

The testing data for loading change detection was formed by concatenating each two load condition signals with fixed other relevant variables. We use the *L*→L+ΔL to represent a loading status change, where *L* hp is the initializing load and ΔL hp is the changing load. More specifically, ΔL>0 represents an increasing load change and ΔL<0 represents a decreasing load change. The combination of L={0,1,2,3} hp and ΔL={−3,−2,−1,1,2,3} hp (indicating different loading conditions in the employed CWRU data set) are used to simulate the loading changes. For example, when the load *L* begins at 3 and decreases by ΔL of −3, the change can be expressed as 3 → 3+(−3). In total, we have 12 combinations of simulated working load changes, as given in Table 1. In each combination, the vibration signals collected from drive end and fan end are used for analysis, respectively; thus, 24 signals are used for change detection.

**Figure 3:**
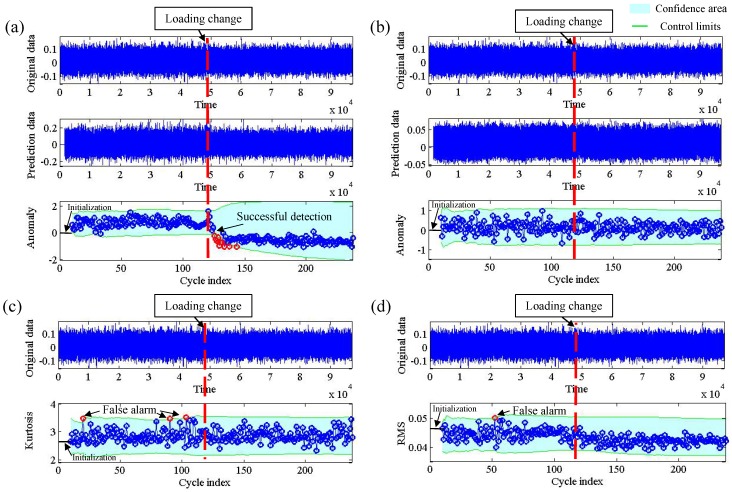
An example of change detection of load condition change from 1 hp to 2 hp. (**a**) DE model; (**b**) ARIMA model; (**c**) kurtosis; (**d**) RMS.

Figure 3 shows an example of change detection for a working load change from 1 hp to 2 hp, where the original signal was collected from drive end with 1:10 down sampling and the change is labeled by a human instructor. From Figure 3a–d, we show the detection result by our method, ARIMA model, Kurtosis and RMS. It can be seen that our method can successfully detect the change cycle with a slight time delay. As seen in Figure 3a, the anomaly scores are relatively small and are distributed irregularly when the motor load is set around 1 hp. Then, the occurrence of loading status change causes an abrupt increase of the anomaly score which can be successfully detected through the 3σ criterion based hypothesis testing; however, the method using the ARIMA model did not detect any alarm because the anomaly score is always in the confidence area, as shown in Figure 3b; the detected change cycle using the Kurtosis and RMS are much earlier than the actual change cycle, which belong to false detection as seen in Figure 3c,d; the main reason is that the proposed method has better power to detect weak changes than the other three methods.

Table 2 gives the comparison results. In the table, the N/3 represents that the number of samples *N* in which change cycle is correctly detected in the three samples, N∈{1,2,3}. It can be clearly observed that our method achieves the best performance and the detection precision is 100%. In other words, whether the vibration signal is collected from the driven or the fan end, the proposed method can detect all the state changes precisely without generating any false alarms.

### 5.2. Case Study II: Bearing Health Status Monitoring

This section considers two real-engineering scenarios of bearing health status: a gradually degenerated process and a sharply degenerated process. Consequently, this case study is employed for this investigation.

The testing data set comes from the publicly available PRONOSTIA platform [34]. This system is designed to test and validate bearings fault detection, diagnostic, and prognostic approaches. The main purpose of PRONOSTIA platform is to provide real experimental data that characterize the degradation of ball bearings along their whole operational life (until their total failure). In order to reduce the bearing’s life duration, a radial force is set to the bearing’s maximum dynamic load of 4 kN and applied on the tested bearings. The force is generated by a cylinder pressure, and the pressure is delivered through a pressure regulator.

During the tests, accelerometers are fixed on the outer race of the bearing, and vibration signals are captured. The sampling rate is 25.6 kHz, the length of every sampled acceleration waveform is 2560 data points, i.e., 0.1 s, whereas recordings are repeated every 10 s. The vibration signals are transmitted into a PC for data visualization and storage through a National Instruments data acquisition (DAQ) card. Based on experience, it is considered as the end of life when the vibration level is greater than 20 g.

The experiment is carried out under three different operating conditions. In our case study, four representative bearing signals during the first operating condition are used for bearing early failure detection. The operating condition is under 1800 r/min (resolution per minute) with a 4000 N load.

**Figure 4:**
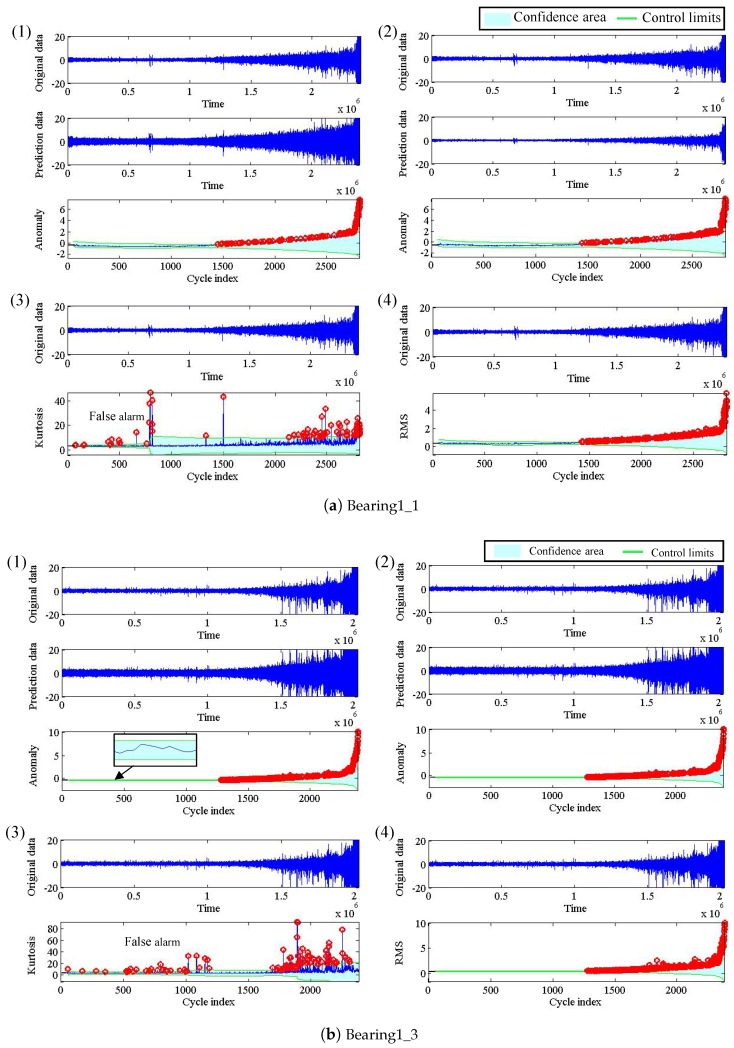

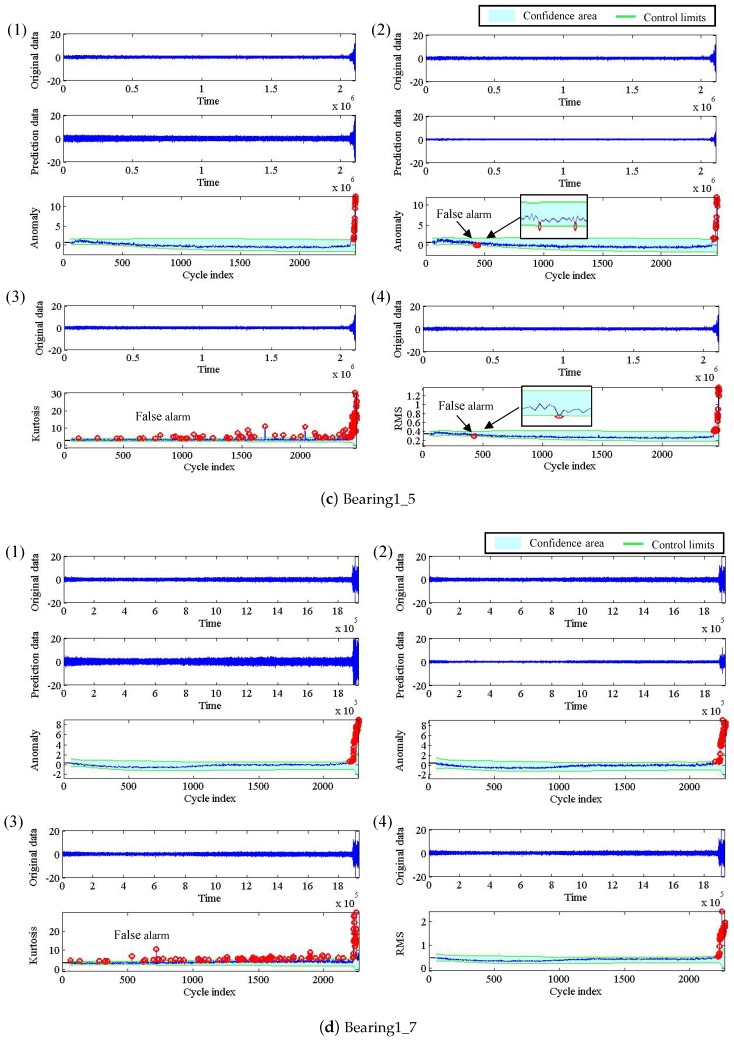
Results of change detection of bearing early failure detection: (**a**) bearing 1; (**b**) bearing 2; (**c**) bearing 3; (**d**) bearing 4. In each piece of testing data: (**1**) the result by DE model; (**2**) the result by ARIMA model; (**3**) the result by Kurtosis, and (**4**) the result by RMS.

Figure 4 shows detection results of the four bearings with different methods. From the original data of each Figure 4a,b, we can see that the vibration amplitude of bearing 1 and bearing 2 have gradually increasing trends, which indicates that the failure gets severe gradually. On the contrary, the vibration amplitude of bearing 3 and bearing 4 increases sharply at the end of lifetime as seen in the original data of each Figure 4c,d, which means a quick degradation processes. Table 3 gives the specific comparison results accordingly, where the number in the table represents the first early failure alarm point, and “N/A” represents false detection status change because there are too many alarm points and the first alarm point is far earlier than the early failure point. Thus, it can be clearly seen that kurtosis fails to distinguish the normal and the abnormal states, which can be verified by subfigure (3)s in all subfigures a, b, c and d in Figure 4. In addition, for bearing 3, as shown in subfigure (c), ARIMA and RMS have false alarms in a normal state. To summarize, the detection performance order is: our method > RMS > ARIMA > Kurtosis, revealing that our method has a superior ability compared to the others.

### 5.3. Case Study III: Speed Condition Monitoring

**Figure 5:**
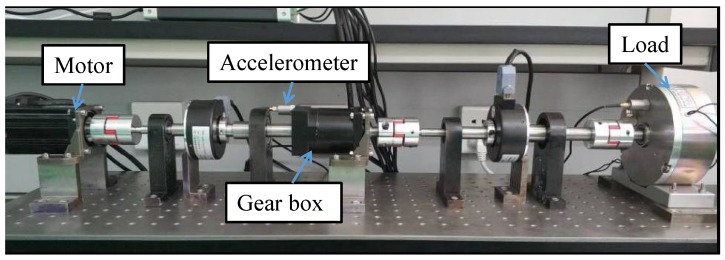
Experimental setup.

We used the experimental setup as shown in Figure 5 to collect testing data. In this setup, an accelerometer sensor mounted on the gearbox was used to acquire vibration signals with a sampling frequency of 1000 Hz, and then the collected signal data were transmitted to PC. During the data collection, we first set the initial speed of motor at ν, and then changed with an interval of Δν to simulate a speed status change (i.e., ν→ν+Δν). The testing values of ν and Δν were given as follows:

• νϵ{250,300,350} rpm and Δνϵ{50,100,150,200,250} rpm.

Therefore, we can obtain 15 parameter combinations with different speed condition changes as seen in Table 4. For each combination, we collected 10 data samples to form testing data set. Thus, we have a total number of 150 data sequences in our testing dataset.

**Figure 6:**
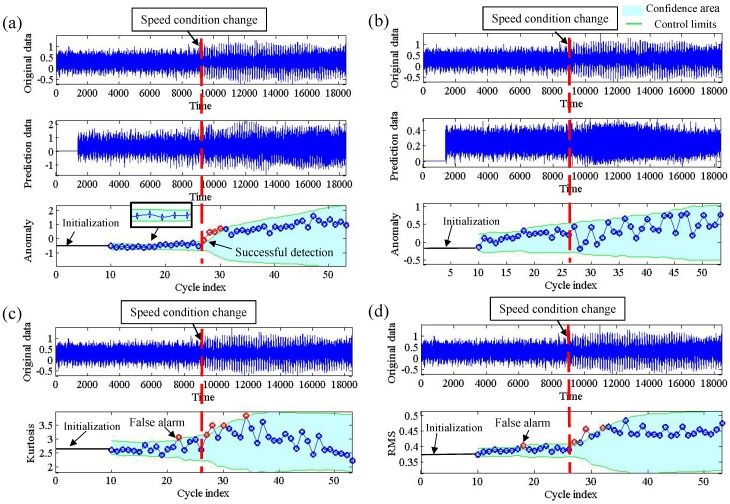
An example of change detection of speed condition change from 350 rpm to 400 rpm. (**a**) DE model; (**b**) ARIMA model; (**c**) Kurtosis; (**d**) RMS.

Figure 6 shows an example of change detection for a speed change from 350 rpm to 400 rpm, where the change is labeled by a human instructor. From Figure 6a–d, we show the detection result by our method, ARIMA model, Kurtosis and RMS. It can be seen that our method can successfully detect the change cycle. As seen in Figure 6a, the anomaly sores show a remarkable increase at the change time such that the change was detected successfully. However, the method using the ARIMA model did not detect any alarm because the anomaly score is always in the confidence area, as shown in Figure 6b; the detected change cycle using the Kurtosis and RMS is much earlier than the actual change cycle, which is attributed to false detection, as seen in Figure 6c,d.

Table 5 gives the comparison results of the different methods under different settings of initial speed ν rpm. We can see that the proposed method also achieves a perfect detection performance on speed status change detection.

### 5.4. Results Summary and Discussion

To summarize, from the above experiments, we notice that the proposed differential equation-based prediction model has prior and remarkable detection performances in three different real applications, especially in weak state change. However, in the proposed method, we assume that the collected condition monitoring signal is periodically stationary. Although this assumption is reasonable and widely used in the literature [7,18,41], we have also noted that the actually collected signal in some cases may vary in periods, i.e., cycle non-stationary. With the purpose of extending the method to these cases, we would employ a preprocessing, e.g., angle re-sampling [55] and phase estimation using dynamic time warping [56,57], in order to suppress such temporal non-stationary before applying it to monitoring non-stationary condition signals.

## 6. Conclusions

In this paper, we have proposed a new method for monitoring and diagnosing the running condition of considered rotating machines. The base of the method is an early detection of condition changes based on the DE model that can realize one-step-ahead prediction of machine status. Together with this model, an analysis of continuously monitoring condition signal by means of sum residual error is presented to inspect/diagnose whether an abnormal condition change occurs or not based on a null hypothesis testing. The method was evaluated with three representative condition monitoring applications: external load status monitoring, bearing health status monitoring and speed condition monitoring. The method was also compared with those benchmark methods reported in the literature. The results demonstrated significant improvements of the proposed method over others, indicating its superiority and great potential in real engineering applications.

In the future, we will optimize the method to achieve a higher computational efficiency, and apply the method in the workshop for practical usages.

## Figures and Tables

**Table 1:** Simulated working load change of *L*→L+ΔL hp.

L\L+ΔL	0	1	2	3
0	-	0→0+(1)	0→0+(2)	0→0+(3)
1	1→1+(−1)	-	1→1+(1)	1→1+(2)
2	2→2+(−2)	2→2+(−1)	-	2→2+(1)
3	3→3+(−3)	3→3+(−2)	3→3+(−1)	-

**Table 2:** Results of loading change for different setting initial working loads *L*.

*Case*\*L*		0	1	2	3	Average
Our method	Drive	3/3	3/3	3/3	3/3	100%
	Fan	3/3	3/3	3/3	3/3	
ARIMA	Drive	3/3	2/3	3/3	2/3	87.5%
	Fan	3/3	2/3	3/3	3/3	
Kurtosis	Drive	3/3	1/3	0/3	0/3	20.8%
	Fan	0/3	0/3	2/3	0/3	
RMS	Drive	1/3	1/3	1/3	1/3	33.3%
	Fan	0/3	0/3	1/3	3/3	

**Table 3:** Results of bearing early failure detection.

Method\Case	Gradual Degeneration		Sharp Degeneration	
	Bearing 1	Bearing 2	Bearing 3	Bearing 4
Our method	1441	1183	2433	2190
ARIMA	1435	1282	424	2184
Kurtosis	N/A	N/A	N/A	N/A
RMS	1422	1083	424	2208

**Table 4:** Simulated speed condition change ν→ν+Δν rpm.

Δν\ν	250	300	350
50	250→250+50	300→300+50	350→350+50
100	250→250+100	300→300+100	350→350+100
150	250→250+150	300→300+150	350→350+150
200	250→250+200	300→300+200	350→350+200
250	250→250+250	300→300+250	350→350+250

**Table 5:** Results of speed condition change detection by different setting initial speeds ν rpm.

	250	300	350	Average
Our method	100%	100%	100%	100%
ARIMA	90%	70%	90%	83.3%
Kurtosis	72%	86%	82%	80%
RMS	100%	44%	80%	74.6%
